# Detecting Pneumonia Using Convolutions and Dynamic Capsule Routing for Chest X-ray Images

**DOI:** 10.3390/s20041068

**Published:** 2020-02-15

**Authors:** Ansh Mittal, Deepika Kumar, Mamta Mittal, Tanzila Saba, Ibrahim Abunadi, Amjad Rehman, Sudipta Roy

**Affiliations:** 1 Department of Computer Science & Engineering, Bharati Vidyapeeth’s College of Engineering, New Delhi 110063, India; anshm18111996@gmail.com (A.M.); deepika.kumar@bharatividyapeeth.edu (D.K.); 2 Department of Computer Science and Engineering, G. B. Pant Government Engineering College, New Delhi 110020, India; mittalmamta@gbpec.edu.in; 3 Artificial Intelligence & Data Analytics (AIDA) Lab, College of Computer and Information Sciences, Prince Sultan University, Riyadh 11586, Saudi Arabia; iabunadi@psu.edu.sa (I.A.); arkhan@psu.edu.sa (A.R.); 4 PRT2L, Washington University in St. Louis, Saint Louis, MO 63110, USA

**Keywords:** pneumonia, chest X-ray (CXR), simple CapsNet, deep learning

## Abstract

An entity’s existence in an image can be depicted by the activity instantiation vector from a group of neurons (called capsule). Recently, multi-layered capsules, called CapsNet, have proven to be state-of-the-art for image classification tasks. This research utilizes the prowess of this algorithm to detect pneumonia from chest X-ray (CXR) images. Here, an entity in the CXR image can help determine if the patient (whose CXR is used) is suffering from pneumonia or not. A simple model of capsules (also known as Simple CapsNet) has provided results comparable to best Deep Learning models that had been used earlier. Subsequently, a combination of convolutions and capsules is used to obtain two models that outperform all models previously proposed. These models—Integration of convolutions with capsules (ICC) and Ensemble of convolutions with capsules (ECC)—detect pneumonia with a test accuracy of 95.33% and 95.90%, respectively. The latter model is studied in detail to obtain a variant called EnCC, where n = 3, 4, 8, 16. Here, the E4CC model works optimally and gives test accuracy of 96.36%. All these models had been trained, validated, and tested on 5857 images from Mendeley.

## 1. Introduction

**Figure 1:**
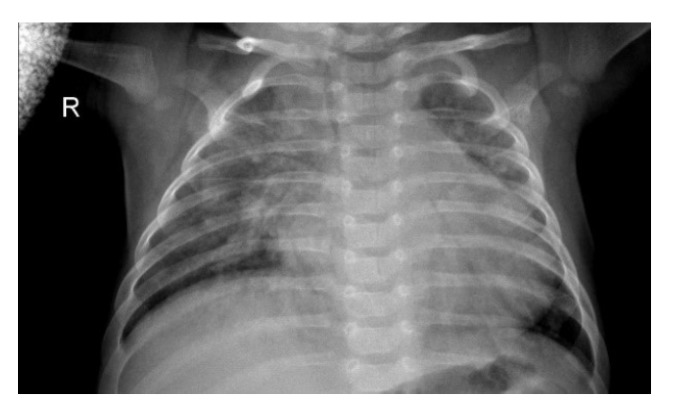
Chest X-ray (CXR) image depicting pneumonia (as can be seen by inflammatory condition of lung).

Pneumonia is one of the most pre-eminent medical diseases in today’s world. A total population of ~450 million (i.e., ~7% of the world’s population) gets diagnosed with pneumonia each year. Of those, an annual fatality rate of approximately 4 million has been observed every year [1]. Therefore, pneumonia remains a leading cause of death for both aged and young in developing countries. Pneumonia can be described as an inflammatory condition of the lung, which affects the small air sacs (more commonly known as Alveoli) present in the organ [2]. The most common clinical method for diagnosis of pneumonia is through chest X-ray (CXR) images. Although this method sounds plausible and helps detect pneumonia better, sometimes even expert radiologists may be frustrated with the conundrum of detecting pneumonia using CXR images (such as shown in Figure 1). This can lead to certain aberrations in the detection of pneumonia which can result in furthering fatalities rather than ameliorating the situation. Therefore, there had been a paradigm shift from manual methods to computational methods.

Some of the earlier works for pneumonia used computer-aided design (CAD) systems for chest radiographs. Even earlier than that, this same task had been accomplished through textual analysis [3,4,5,6]. The same can be inferred from the CAD system at the Kun Rossman Lab, which is located in Chicago [7]. This CAD system divided the lung images into multiple Region of Interest (ROIs) to determine and analyze if any abnormalities existed in the ROIs. These further led to the integration of CAD systems with Deep Neural Network (DNNs) algorithms. This had finally led to the usage of pretrained Convolutional Neural Networks (CNNs) and pretrained networks such as U-Net, CardiacNet, and SegNet for pneumonia detection [8,9,10]. Apart from this, many approaches using reinforcement learning and evolutionary algorithms, to optimize hyperparameters of DNNs, have been introduced. Nevertheless, these approaches have proven to be time-consuming, while also requiring high computation power. This leads to the problem of finding better algorithms for detecting Pneumonia.

Capsule network (more commonly known as CapsNet) [11] has been one of the algorithms that have been leading in generative and deterministic capabilities. These algorithms have been much more sensitive to images than the previously introduced CNNs. These networks, quite literally, squash multiple convolutional layers in capsules which are then subjected to nonlinearity. This is quite similar to what had been done in CNNs for a single neural layer. As CNNs have been used in medical informatics [12,13,14], CapsNet have also been increasingly employed in medical tasks such as brain tumor segmentation and classification [15] and works such as lung cancer detection [16] and blood cell classification [17].

As would be clear later onward, this work’s contribution has been fivefold, to be true to this paper’s title, which has been summarized as follows.

1. A comparative analysis of various deep learning algorithms has been conducted in Table 1. This analysis depicts that among the commonly used model, the Okeke Stephan method provides one of the best accuracies.
2. A simple CapsNet model has been used to check the performance of detecting pneumonia in CXR images. The accuracy for this model is compared to the earlier best accuracy obtained using CNN models.
3. Two other models have been defined for the detection of pneumonia, which perform even better than the aforementioned simple CapsNet.
4. The latter of the model, which performed optimally for pneumonia detection, has therefore been studied in more detail. This was done by creating its variants.
5. All models studied so far have been compared completely in terms of their validation and test accuracies, validation loss and total loss; among these, the most accurate of the variants had been taken as the currently best working model for this task.

This paper has been organized as follows. Section 2 discusses the works done earlier for detecting pneumonia while also comparing these works. Section 3 elucidates the model architectures in detail along with the dataset and the mathematics involved in the research. Section 4 enlists results obtained by all the models that have been described in Section 3. Finally, conclusions have been drawn from the results obtained and briefly discussed along with the limitations in the research conducted.

## 2. Related Works

Earlier works of pneumonia detection mainly dealt with CAD systems (as aforementioned). These works gave low accuracy in comparison to DNNs and CNNs, which were introduced at a later time. Pneumo-CAD [18] had been a state-of-the-art CAD system at that time. This system classified if someone had a presence of pneumonia or no presence of it in the CXR images. This classification worked by assigning two different classes—PP (Pneumonia Present) or PA (Pneumonia Absent)—under the CAD system. Its design utilized a k-Nearest Neighbor (kNN) scheme and/or Haar wavelet transforms. These methods were able to achieve considerable results and had a comparatively low misclassification rate of ~20% with kNN, and 10% with kNN and Haar Wavelet, despite the area-under-curve (AUC) value being comparatively less for receiver operating characteristic (ROC) curve. Later on, a comparative analysis of the algorithm had been conducted using Pneumo-CAD with Sequential Forward Elimination (SFE), Pneumo-CAD without SFE, and Support Vector Machine (which used SFE) gave accuracies of 66%, 70%, and 77% [19], respectively. This research also tested the Naïve Bayes algorithm and got an accuracy of 68% with it.

A comparative study performed in [20] and works done in [21] (such as the ones that used Self Organizing Maps (SOM) (This research was performed by Moh’d Rasoul, A and Dorgham, O and Razouq, Rami Salim in work “Pneumonia identification using organizing map algorithm” in 2006.) and Fuzzy C-Means) had been performed for finding the optimal model for detecting pneumonia. The algorithms both have pretrained weights (from datasets such as ImageNet [22]) and are user-defined, and have also been extensively used for detecting pneumonia. This can be inferred from the works done by Pranav Rajpura et al. [23] and Can J Saul et al. [24]. CheXNet, introduced in the former work, utilized 121 layer CNN that had been trained on ChestX–ray14. Despite the algorithm providing a relatively low accuracy of approximately 76.80% for predicting pneumonia, it had been able to classify 13 other chest diseases from CXR images. The latter researchers proposed a CNN architecture for early diagnosis of pneumonia and achieved an accuracy of 78.73% with a much simpler architecture. Apart from these studies, there have been works [25,26] that utilized Xception, VGG16, and VGG19 models [27,28] that were pretrained on the ImageNet dataset. These works managed to achieve an accuracy of 82% for the Xception pretrained model, 87% for the VGG16 model, and 92% for the VGG19 model. The authors of [29] compared the proposed CNN model architecture with algorithms such as Backpropagation Neural Networks (BPNN), Competitive Neural Networks (CpNN), and pretrained and fine-tuned networks (like VGG16 and VGG19). Later, Okeke Stephan et al. [30] succeeded in achieving a validation accuracy of 93.73% for similar classification with the usage of a user-defined CNN architecture.

Other than these attempts at detecting pneumonia, there had been many more attempts to detect pneumonia accurately. Nonetheless, all these attempts have not been able to outperform algorithms described in work done by Okeke Stephan et al. Here, the first attempt had been made by V. Martinez using ResNet-152 (This work was done in article “Detecting Pneumonia in Chest X-Rays with Custom Vision and PyTorch”.). This model gave an accuracy of approximately 88%. The second attempt had been made by A. Imran, using another VGG16 model, which achieved an accuracy of approximately 88.89% (This work was introduced in article “Training a CNN to detect Pneumonia”.), whereas the third attempt by A. Sagar had been made using CNN architecture where batch normalization after every convolutional layer, had been conducted (Work introduced in article “Deep Learning for Detecting Pneumonia from X-ray Images”.). This model simultaneously reduced the learning rate of the model if there was no decrease in validation loss. This work achieved a validation accuracy of 91.55% and a test accuracy of 91.02%. All these attempts were made on a very famous and commonly used pneumonia dataset CXR image dataset known as chest X-ray (CXR) images [31] published in Mendeley. Other attempts have also been made that could utilize ResNet34 to get an accuracy of approximately 91% (This was conducted by Tian, Y.(2018) in article "Detecting Pneumonia with Deep Learning.”) and the next attempt by J. Erthal got an accuracy of approximately 80% (This work had been introduced in article “Detecting Pneumonia with Deep Learning: A Soft Introduction to Convolutional Neural Networks”.).

Recently, there has been a shift from CNN models to the more compact and hierarchical capsule networks (h-CapsNet). This had been made possible due to the early works in the fields of capsules networks, starting with work done in [11] and then advancing in [32,33,34,35]. There have been many fields that utilize CapsNet to get better results that were earlier not possible using DNNs, CNNs, or any other classification algorithm. These fields have been inclusive of brain tumor segmentation, lung cancer classification, and blood cell classification. Apart from this, CapsNet had recently been used for detecting pneumonia along VGG16 blocks to create an integrated model (This algorithm was used by Cardoso, R. in article “Using VGG + CapsNet in to Diagnose Pneumonia | Kaggle”.). Even after this integration of a pretrained VGG16 and state-of-the-art algorithm, the algorithm had only been able to achieve a validation accuracy of 87.5% for the training rate of 97.18%. Despite this, the model had been able to achieve a testing accuracy of 88.3%. Therefore, we see that CapsNet models have been much more reliable and sensitive to the detection of pneumonia from CXR images.

A brief analysis of all the algorithms mentioned in the literature has been conducted. This had been done in terms of the accuracy they achieved on the CXR image dataset or ChestX-ray14 dataset (used in the case of CheXNet). This analysis has been mentioned below in Table 1.

**Figure 2:**
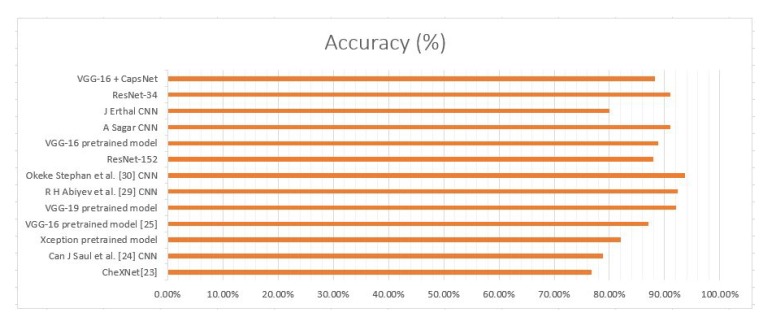
Pneumonia detection using chest X-ray (CXR) image through deep learning models in accordance with Literature.

A further extrapolation of Table 1 has been done in Figure 2 given below. It shows that although the combination of VGG16 and CapsNet model had been able to get accuracy of just 88.30%, it can still perform better as the model had been run on just 10 epochs. Therefore, this depicts that CapsNet has a lot of potential to more accurately detect pneumonia.

## 3. Proposed Methodology

### 3.1. Dataset Description

**Figure 3:**
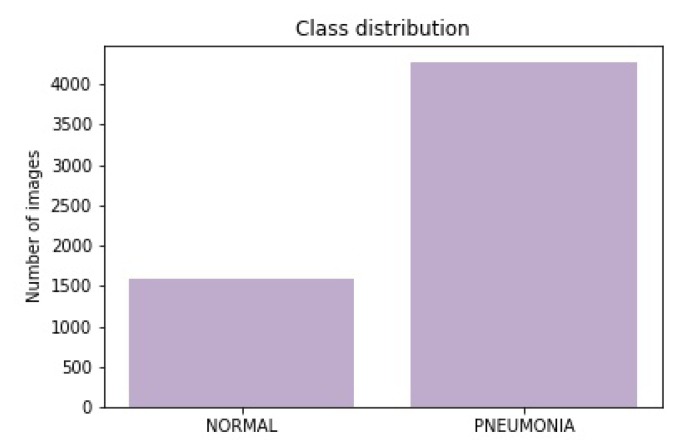
The complete class distribution of the complete CXR image dataset used to train, validate, and test the model mentioned in this work.

The dataset consists of CXR images that were taken from the Mendeley CXR image dataset [31]. This dataset consists of 5857 CXR images. These have been divided into 2 classes—“Normal” and “Pneumonia”. The distribution (i.e., classes and training, validation, and testing set) for these images has been depicted below in Table 2, whereas the class distribution of the dataset has been shown in Figure 3.

**Figure 4:**
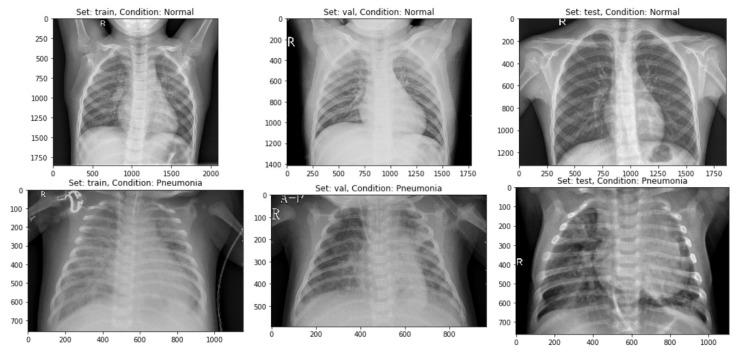
A sample of CXR images having both the classes separated into Train, Validation, and Test datasets.

This depicts that there were 1583 images for the “Normal” class and 4274 images for class “Pneumonia”. This depicts that ~27% of the data had been represented through “Normal” class, whereas ~63% of the CXR images had “Pneumonia”. As the CXR images can be sensitive to slight changes in shearing, rotation, or zooming, data augmentation had been avoided in the CXR image dataset. Therefore, the data could not be undersampled or oversampled. The sample of images for each of the sets has been displayed below as in Figure 4.

### 3.2. Regularization Techniques

#### 3.2.1. Dropouts

To reduce overfitting, the third model and its variants (that have been introduced later onward) have utilized regularization techniques also known as dropouts, which have been employed after pooling layers in most of the dense convolution subnetworks of the model. Dropout had been set to 0.5 or 0.25 (as illustrated later in Figures 7–9), which depicts that the weights of the half or a quarter of the neurons had randomly been set to zero, respectively, whereas remainder neurons gave original inputs multiplied by the corresponding weights. This ensures that the neurons had been independent of the other neurons.

#### 3.2.2. Decoder Network

**Figure 5:**
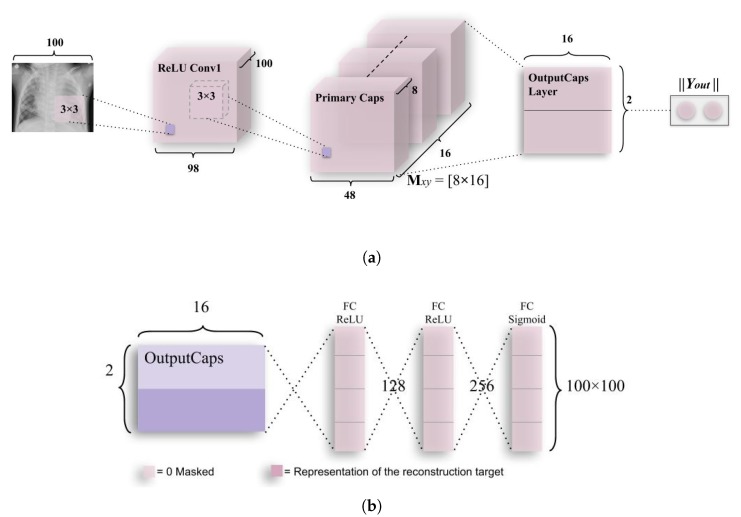
The architecture of capsule networks along with the decoder module. (**a**) A simple CapsNet model achieves accuracy that is comparable to best CNN till now. The length of class instantiation activity vector of “Normal” and “Pneumonia” classes in output capsule layer indicates presence of one of the class (in a specific CXR image), which is used to calculate classification loss. As discussed above, weight matrix $M_{xy}$ is a matrix of weights between both $\alpha_{x}$, x∈ (1, 16 × 48 × 48) in Primary Caps layer and$v_{y}$, y∈ (1,2), where 1 represents “Normal” and 2 represents “Pneumonia”. (**b**) Decoder structure for reconstruction of CXR images from the class representation. The mean square error is minimized during the training phase of the network. The decoder has comparatively subservient role to the CapsNet in this research as it is used for encoding and regularizing the activity instantiation vector. In this research, the decoder will mostly be stuck in a local optimum due to less no. of capsules mapping values to the reconstruction layer, which leads to a lower accuracy of the image.

This network comprises DNN layers to generate back the pixel intensities using the output from the activity instantiation vector from each class in the CapsNet or other models. There have essentially been two types of decoders that have been used in this research. One of the decoder networks has been depicted in Figure 5b and Figure 9b. These decoder layers also work as regularizing layers in the decoder networks. First, these decode the activity vector and then map the best weights through which these vectors could have been decoded. This is then used in all models (except that of Simple CapsNet) to get back the convoluted feature maps of the original image. These weights of convoluted feature maps are backtracked and augmented to each convolutional layer, therefore reducing overfitting and resulting in the regularization of trained models defined throughout this text.

### 3.3. Model Architecture

#### 3.3.1. Simple CapsNet

Initially, a CapsNet [11] architecture had been used (as shown in Figure 5) to check if the model could predict pneumonia on par with the work done in [30], where 93.73% accuracy had been achieved. This simple architecture has only two capsule layers in addition to one convolutional layer. The ReLU Conv1 layer had 100, 3 × 3 kernels of convolutions with single stride for feature vector of 98 × 98. As can be seen from the name of the layer, Conv1 introduces nonlinearity using Rectified Linear Unit (ReLU) activation function [36]. Here, values of local features have been obtained using the pixel intensities of 100 × 100 (resized as the number of parameters exceeded 120 M with 200 × 200 images having RGB channels) CXR images, which have then been used in primary capsules (in the layer, Primary Caps).

**Figure A1:**
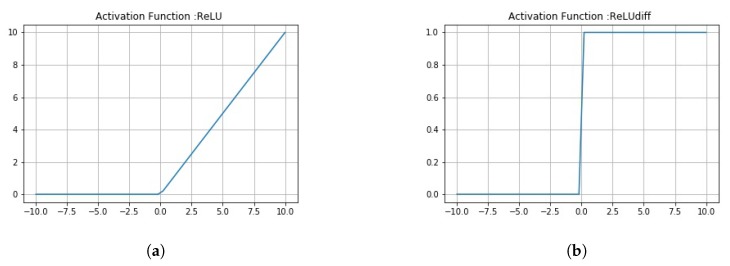
(**a**) A plot of Rectified Linear Unit (ReLU) activation unit function. (**b**) A plot of derivative of ReLU activation unit function.

The function for ReLU has been discussed along with its graph in Figure A1, which had been plotted using Numpy [37] and Matplotlib [38].

The Primary Caps, i.e., the second layer have multidimensional entities that can be placed at the lowest level, hierarchically. This layer when activated is similar to the inverse process of rendering an image. This (second) layer is the first capsule layer having 16 channels for convolution 8-D capsules (so every primary capsule contributes eight units (similar to feature maps in CNNs) with 3 × 3 kernels and 2 as stride). Each of these primary capsules had been provided with 100 × 9 units from ReLU Conv1. The Primary Caps have 16 × 48 × 48 outputs from the capsules (where each output is 8-D). Here, each capsules in a 48 × 48 feature map share its weight with other capsules. For this block, the nonlinearity function used had been provided by the function given below in Equation (1),

(1) $$v_{y}=\frac{|s_{y}{|}^{2}}{1+|s_{y}{|}^{2}}\cdot \frac{s_{y}}{|s_{y}|}$$

where $s_{y}$ is the total input vector while $v_{y}$ is the output vector from capsule *y*. Here, the probability of entity (or class, i.e., “Normal” or “Pneumonia”) being present in the input vector, that capsule represents has been depicted by the length of the capsule. This function ensures that the short vector gets shrunk to approximately zero while a large vector gets shrunk to the length that has been slightly less than unity. This has therefore, been named squashing function [11]. The final layer (output capsule layer (or OutputCaps)) has a 16-D capsule unit for both “Normal” and “Pneumonia” classes (i.e., 16 × 2 matrix). As there have been only two capsule layers (Primary Caps and OutputCaps) that have been juxtaposed, the routing is done between these two. As the output from ReLU Conv1 can be just 1-D, there has been no orientation in ReLU Conv1 in which dynamic routing can be applied. The routing algorithm has been briefly explained as was introduced in [11].

Every capsule layer except the first capsule layer (here, Primary Caps), has $s_{y}$ (of Equation (3)) as a weighted sum over all predictions of entity $\alpha_{y|x}$ from the capsule layer below. The value of $s_{y}$ is calculated by multiplying output $\alpha_{x}$ of the capsule by the matrix of weights $M_{xy}$. This has been depicted in Equation (2) below.

(2) $$s_{y}=\sum_{x}k_{xy}\cdot a_{y|x},\;\mathrm{where},\;a_{y|x}=M_{xy}\cdot \alpha_{x}$$

where iterative dynamic routing can be described using coupling coefficients, $k_{xy}$. The coupling coefficients between capsules above the layer having capsule *x* and itself sum significantly above unity which has been determined by softmax (which can therefore be called routing-softmax, and has been given in Equation (3)) whose initial class units $b_{xy}$ are log a priori probabilities that capsule *x* and capsule *y* have been coupled.

(3) $$k_{xy}=\frac{e^{b_{xy}}}{\sum_{s=1}^{S}e^{b_{xs}}}$$

The log a priori values are learned simultaneously as all other weights. This results in them being dependent on locations and type of two capsule layers instead of the current CXR image (i.e., even if current CXR image is in a different orientation, these routings work robustly). This suggests that the CapsNet model follows equivariance instead of invariance for the orientation of objects in the images. The routings work by first initializing all the class units to zero, after which a capsule output ($\alpha_{x}$) is sent to both parent capsules ($v_{0}$ and $v_{1}$, which are determined by the number of classes) with equal probabilities ($k_{xy}$). The final model had been created using Keras [39] and Tensorflow API [40]. The compilation of this model had been done using RMSProp optimizer [41] for mean square error (MSE) and margin loss (which has been given in Equation (4) below).

(4) $$L_{s}=T_{s}\cdot max(0,m^{+}-|v_{s}{\left|\right)}^{2}+\lambda \cdot \left(1-T_{s}\right)\cdot max(0,|v_{s}{\left|-m^{-}\right)}^{2}$$

As explained earlier, the length of the vector represents the possibility of an entity (or class) to exist in the pre-squashed vector, in a capsule. The top-level capsule, which denotes the output class, has long instantiation vector (in this model, it had been 16-D) iff the entity (or class) represented in the vector can be found in that specific image. As this is a binary classification problem, $T_{S}$ represents if the image is detected as “Pneumonia” or “Normal”, and $m^{+}$ or $m^{-}$ will be 0.5, respectively. The down-weighting factor λ puts an impasse on the shrinking activity vector for “Normal” or “Pneumonia” in case “Pneumonia” or “Normal” have been detected by the receptive fields from the capsules, respectively. Here, the total loss is the combined loss of capsules representing both classes “Normal” and “Pneumonia”. Finally, $Y_{out}$ has been used for determining the probability of a CXR image to be detected as “Normal” or “Pneumonia”. These values as mentioned above have been obtained by the application of Equation (5).

To encourage the class capsules to encode parameters of the input “Normal” and “Pneumonia” CXR images, a reconstruction layer has been added. This has been done to counter the effects of attacks, such as the one performed in [42], where one pixel had been perturbed to disrupt the classification performed by DNNs. Although this had been performed on the CIFAR-10 dataset, such types of security threats if applied on CXR images can cause fatalities in case of detecting pneumonia as when a single pixel is perturbed, it may cause a CXR scan of “Pneumonia”, to be falsely detected as “Normal”. Therefore, while training this layer, all layers except that of the third layer (also, second capsule layer), have been masked. From this layer, receptive fields of activity instantiation vectors are utilized to reconstruct the CXR image and finally, all parameters are backtracked to encode the pixel intensity values, which makes the model more robust to the aforementioned pixel attacks. The output of this layer along with the two classes mentioned earlier, has been fed into decoder (Although the decoder is used to decode the class instantiation vector and generate back the image, the accuracy of the decoder is substantially compromised in this research as the architecture that has been defined for the decoder is not able to accurately predict the intensities for each pixel to generate back an image having 40,000 (200 × 200) pixels for RGB values each (total pixel values is 120,000).) consisting of fully connected layers that model pixel intensities, as has been depicted in Figure 5b. The sum of squared differences between logistic unit output, and intensity of pixels of the actual image is minimized by a very small amount; therefore, the margin loss is not obscured during this process. This is because the main task of this network is to detect pneumonia, rather than to work as a generative model.

**Figure A2:**
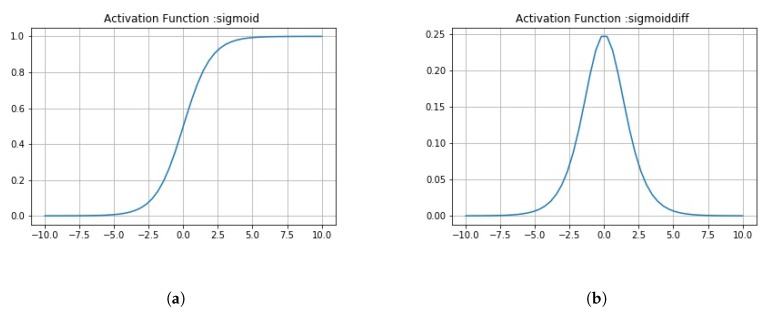
(**a**) A plot of ReLU activation unit function; (**b**) A plot of derivative of ReLU activation unit function.

This encoding-cum-regularizing layer has been depicted above in Figure 5b. The first two fully connected layers use the ReLU nonlinearity function which has been explained using Equations (A1) and (A2) and Figure A1a,b. These layers have 128 and 256 neurons, respectively. They take an activity instantiation vector of 16 × 2 (where 2 is no. of classes), which is given from the third layer (Output Caps) of the CapsNet architecture explained above. In addition to this, another 2-D vector, that states, if the class is detected as “Normal” or “Pneumonia”, has been augmented to the activity instantiation vector. Finally, the last fully connected layer gives the output image using sigmoid function [43] that has been discussed along with its graph in Figure A2.

#### 3.3.2. ICC (Integration of Convolutions with Capsules)

**Figure 6:**
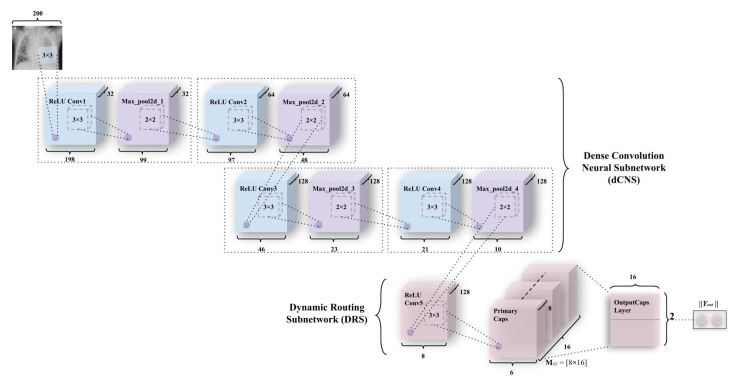
An integration of convolutions and capsules, which has been named Integration of convolutions with capsules (ICC). This model outperforms earlier simple capsule model (in Figure 5a). As discussed earlier, weight matrix $M_{xy}$ has been a matrix of weights between both $\alpha_{x}$, x∈ (1, 16 × 6 × 6) in Primary Caps layer and $v_{y}$, y∈ (1,2), where 1 represents “Normal” and 2 represents “Pneumonia”.

The second model—integration of convolutions with capsules (ICC), depicted in Figure 6—used for detecting pneumonia, is a combination of convolutions with capsule layers among which (simple capsules at the bottom of Figure 6), the routing occurs. This network has two subnetworks—the dense convolution neural subnetwork (or also known as dCNS) and the dynamic routing subnetwork (or also known as DRS).

The dCNS in the ICC model consists of four maxpool layers corresponding to each of the four convolution layers present in the dCNS. These convolutions uniformly introduce nonlinearity using the ReLU activation function (described in Equations (A1) and (A2) and Figure A1a,b using a kernel of size 3 × 3, whereas maxpool is uniformly imposed on pool size of 2 × 2. The ReLU Conv1 layer has 32 feature maps that utilize 198 × 198 feature vectors from these, which have later been subjected to maxpooling to get 99 × 99 feature vectors. These two layers have been integrated into one block for the simplicity of representation, as has been depicted in Figure 6. This process has been repeated through ReLU Convolutional layers and Max_pool2d layers (through ReLU Conv2, Max_pool2d_2, ReLU Conv3, Max_pool2d_3, ReLU Conv4, Max_pool2d_4) to obtain feature maps of vectors having 64 × 97 × 97 and 64 × 48 × 48 (2nd convolution-maxpool block), 128 × 46 × 46 and 128 × 23 × 23 (3rd convolution-maxpool block), and 128 × 21 × 21 and 128 × 10 × 10 (4th convolution-maxpool block) in the same order.

The DRS—the other part of the ICC—consists of the simple CapsNet explained earlier, albeit, with a shrunken activity instantiation vector due to earlier convolutions on the CXR images. Here, ReLU Conv5 consists of feature vectors of dimensions 8 × 8. This is then mapped to the Primary Caps layer which has 16 channels of 8-D capsules (so every primary capsule contributes 8 units, with 3 × 3 kernels and 2 strides). Each of these primary capsules has been provided with 128 × 9 units from the ReLU Conv5 layer. The Primary Caps have 16 × 6 × 6 outputs from the capsules (where each output is 8-D). Here, each capsule in a 6 × 6 feature map shares their weight for equivariance to the perceptive fields of capsules. For this block, the nonlinearity remains the same as has been provided by the function in Equation (3). Apart from this, all the configuration for the capsule layers (i.e., their routing, their margin loss, etc.) remains as had been used in the simple CapsNet model. Finally, $Y_{out}$ has been used for determining the probability of a CXR image to be detected as “Normal” or “Pneumonia”.

As mentioned in the simple CapsNet model, to promote the class capsules for encoding parameters of the CXR “Normal” and “Pneumonia” image, a reconstruction layer has been added. The reason for the encoding of these activity instantiation vectors here is quite different. As explained earlier, in the simple Capsnet model, the use of a decoder network makes it easy to map the activity instantiation vector to pixel intensities of an input image. However, here the input to decoder is the convoluted image pixels that went through a concatenation. Therefore, here the decoder network gives the accuracy for the reconstructed image being the actual image (but here reconstructed image is the convoluted pixel values). Thus, here and in the next model encoding, is applied to convoluted features that further backtracks the encodings back through the convolutional layer. This results in a function similar to introducing regularization through capsule layers as well as convolutional layers (apart from the dropout values). Therefore, the encoding-cum-regularization-cum-reconstruction layer depicted in Figure 5b remains the same for the ICC model, but performs more important functions such as regularizing the whole convolution functions. This helps increase the accuracy of the model. This model has been inspired by models such as [44,45], which utilize capsules in conjunction with convolutions with or without the addition of feedback, respectively.

#### 3.3.3. ECC (Ensemble of Convolutions with Capsules)

**Figure 7:**
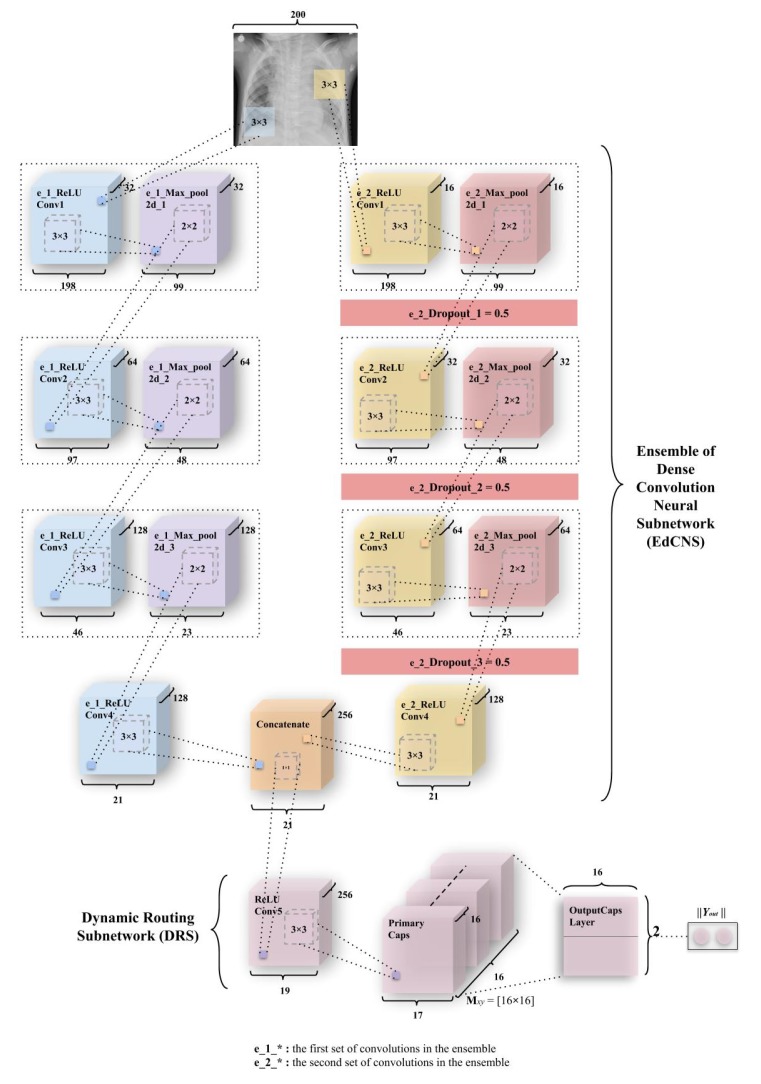
An integration of convolutions and capsules which has been named Ensemble of convolutions with capsules (ECC). This model outperforms earlier simple CapsNet model (in Figure 5a) and comparable to ICC (depicted in Figure 6). As discussed earlier, weight matrix $M_{xy}$ is a matrix of weights between both $\alpha_{x}$, x∈ (1, 16 × 17 × 17) in Primary Caps layer and $v_{y}$, y∈ (1,2), where 1 represents “Normal” and 2 represents “Pneumonia”.

The third and the final model—Ensemble of convolutions with capsules (ECCs) presented in Figure 7—is a combination of ensemble of convolutions. This network also has two subnetworks—the ensemble dense convolution neural subnetwork (known as EdCNS) and the dynamic routing subnetwork (known as DRS).

The EdCNS in the ECC model consists of 8 convolutions to which, the 6 maxpool layers correspond to (divided among two ensembles of 4 convolutions and 3 maxpooling). These convolutions uniformly retain the configuration (nonlinearity, kernel size, and pool size) from the ICC model. The e_1_ReLU Conv1 (Note: (e_1_) refers to the first set of convolutions in ensemble, and (e_2_) refers to the second set of convolutions in ensemble.) layer has 32 feature maps, which have 198 × 198 feature vectors from the input images that have been then subjected to maxpooling to get 99 × 99 feature vectors. Figure 7 combines both of these layers into one block as had been done in ICC. This process is repeated through ReLU Convolutional layers and Max_pool2d layers (through e_1_ReLU Conv2, e_1_Max_pool2d_2, e_1_ReLU Conv3, and e_1_Max_pool2d_3) to obtain feature maps of vectors having 64 × 97 × 97 and 64 × 48 × 48 (2nd block of convolution-maxpool), 128 × 46 × 46 and 128 × 23 × 23 (3rd block of convolution-maxpool), and finally, 128 × 21 × 21 through e_1_ReLU Conv4. In the second set of convolutions, the e_2_ReLU Conv1 layer has 16 feature maps that utilize 198 × 198 feature vectors from an input image, which have been subjected to maxpooling to get 99 × 99 feature vectors. Then, a dropout (e_2_Dropout_1) of 0.5 is applied (as had been mentioned in Section 3.2.1). The Figure 7 combines all three convolutional, maxpool, and dropout layers (through e_2_ReLU Conv2, e_2_Max_pool2d_2, e_2_Dropout_2, e_2_ReLU Conv3, e_2_Max_pool2d_3, and e_2_Dropout_3) to obtain feature maps of vectors having 32 × 97 × 97 and 32 × 48 × 48 (2nd block of convolution-maxpool-dropout), 64 × 46 × 46 and 64 × 23 × 23 (3rd block of convolution-maxpool-dropout), and conclusively 128 × 21 × 21 using e_2_ReLU Conv4, in order. The output from the e_1_ReLU Conv4 and e_2_ReLU Conv4 is merged using the “concatenate” function of the Keras package similar to the work [46], where instead of concatenating ReLU units, the model concatenated different feature maps and dropouts.

#### 3.3.4. EnCC (Ensemble of n-Convolutions with Capsules)

**Figure 8:**
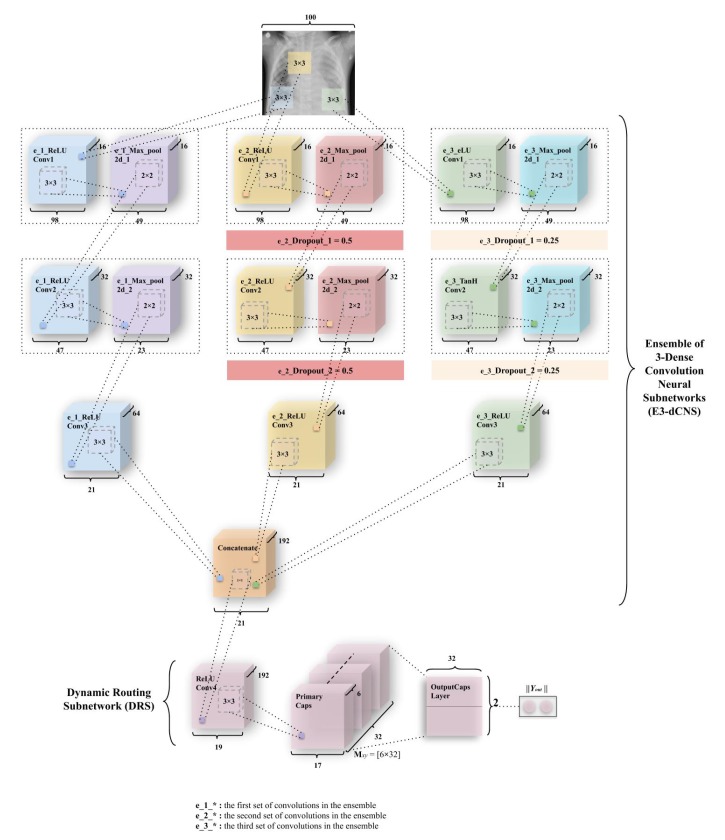
A variant of ECC, named Ensemble of 3-convolutions with capsules (E3CC). As discussed earlier, weight matrix $M_{xy}$ is a matrix of weights between both $\alpha_{x}$, x∈ (1, 32 × 17 × 17) in Primary Caps layer and $v_{y}$, y∈ (1,2) where 1 represents “Normal” and 2 represents “Pneumonia”.

**Figure 9:**
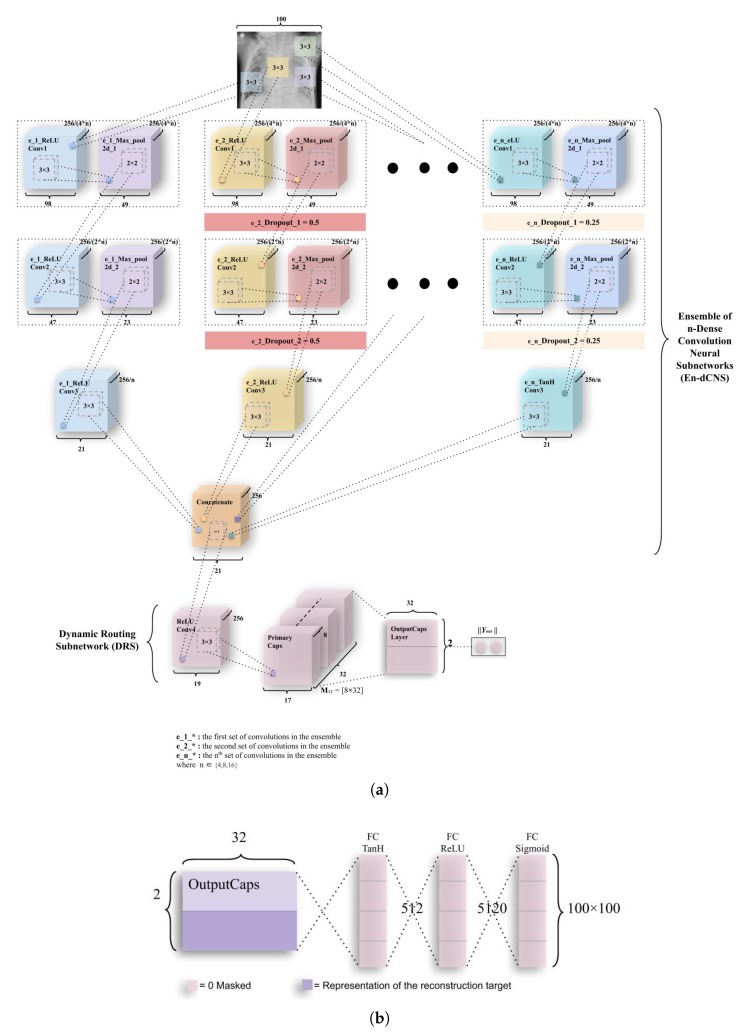
The architecture of complete EnCC networks along with the decoder module. (**a**) A variant of ECC, named Ensemble of n-convolutions with capsules (EnCC). As discussed earlier, weight matrix $M_{xy}$ is a matrix of weights between both $\alpha_{x}$, x∈ (1, 32 × 17 × 17) in Primary Caps layer and $v_{y}$, y∈ (1,2) where 1 represents “Normal” and 2 represents “Pneumonia”. (**b**) Decoder structure for reconstruction of CXR images from the class representation. The mean square error is minimized during the training phase of the network. The decoder has comparatively subservient role to the CapsNet in this research as it is used for encoding and regularizing the activity instantiation vector. In this research, the decoder will give low values as input image is compared with the convoluted images, which give mapping values to the reconstruction layer, thus leading to a lower accuracy of the image.

The variant of the third model—Ensemble of n-convolutions with capsules (ECCs) presented in Figure 8 and Figure 9—is a combination of the ensemble of convolutions similar to ECC, except for the more number of convolutions subnetworks that have been introduced in these models. This network, like ECC, has two subnetworks—the ensemble n-dense convolution neural subnetwork (known as En-dCNS) and the dynamic routing subnetwork (known as DRS). Here, n ∈ (3,4,8,16). For the variants introduced in this section, the image size had been resized to 100 × 100 so the number of features could be reduced by a factor of 4.

**Figure 10:**
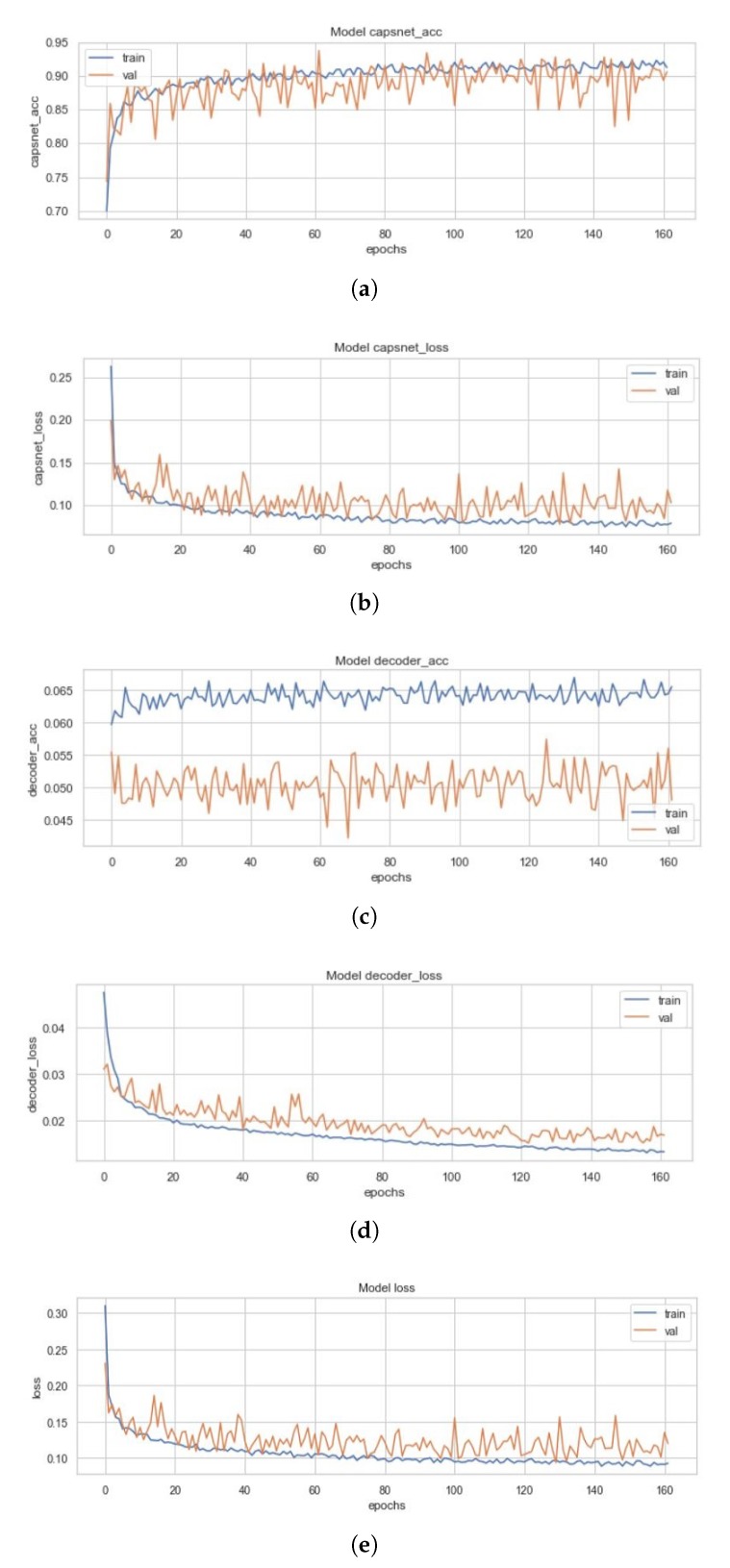
(**a**) Validation (in orange) and Training Accuracy (in blue) during the training of CapsNet. (**b**) Validation (in orange) and Training Loss (in blue) during the training of CapsNet (**c**). Validation (in orange) and Training Accuracy (in blue) during the training of Decoder (**d**). Validation (in orange) and Training Loss (in blue) during the training of Decoder. (**e**) Validation (in orange) and Training Loss (in blue) during the training of CapsNet + Decoder.

**Figure A3:**
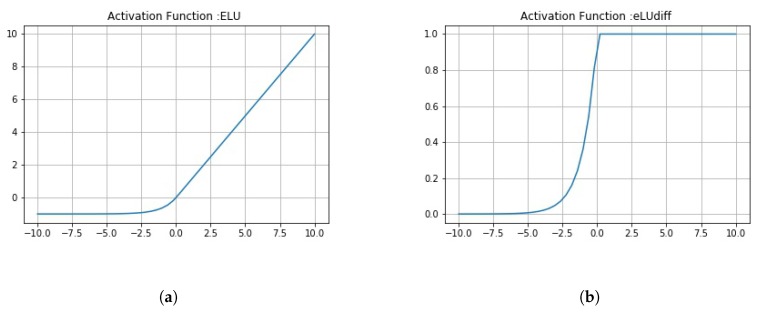
(**a**) A plot of Exponential Linear Unit (eLU) activation unit function; (**b**) A plot of derivative of eLU activation unit function.

**Figure A4:**
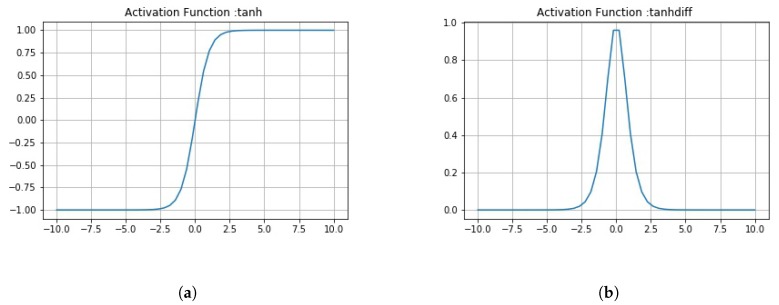
(**a**) A plot of Hyperbolic Tangent (TanH) activation unit function; (**b**) A plot of derivative of TanH activation unit function.

As the E3CC model is different from other variants introduced, it has been explained separately from the rest of the variants. In this variant, the E3-dCNS in the ECC model consists of 6 maxpool layers corresponding to 9 convolutions, which the variant consists of. These are divided among three ensembles of 3 convolutions and 2 maxpooling. The e_1_ReLU Conv1 layer has 16 feature maps that utilize 98 × 98 feature vectors from these, which have then been subjected to maxpooling to get 49 × 49 feature vectors. Figure 10 combines both of these layers into one block as had been done in the actual ECC model. This process is repeated through ReLU Convolutional layers and Max_pool2d layers (through e_1_ReLU Conv2, e_1_Max_pool2d_2) to obtain feature maps of vectors having 32 × 47 × 47 and 32 × 23 × 23 (2nd block of convolution-maxpool), and finally, 64 × 21 × 21 through e_1_ReLU Conv3. In the second subnetwork, the e_2_ReLU Conv1 layer has 16 feature maps that utilize 98 × 98 feature vectors from the reduced input image, which have again been subjected to maxpooling to get 49 × 49 feature vectors. Then, a dropout (e_2_Dropout_1) of 0.5 is applied. Figure 10 combines all three convolutional, maxpool, and dropout layers (through e_2_ReLU Conv2, e_2_Max_pool2d_2, e_2_Dropout_2) to obtain feature maps of vectors having 32 × 47 × 47 and 32 × 23 × 23 (2nd block of convolution-maxpool-dropout), and conclusively 64 × 21 × 21 using e_2_ReLU Conv4, in order. The third subnetwork of convolutions works similar to the second subnetwork. The only difference being that this third subnetwork uses a dropout of 0.25 and 2 different nonlinearity functions—the first one being eLU (exponential Linear Unit), and the second being Tanh (Hyperbolic Tangent). The equations for this nonlinearity and their derivatives have been defined in Figure A3 and Figure A4. The output from the e_1_ReLU Conv3, e_2_ReLU Conv3 and e_3_ReLU Conv3 is merged using “concatenate” function.

**Figure 11:**
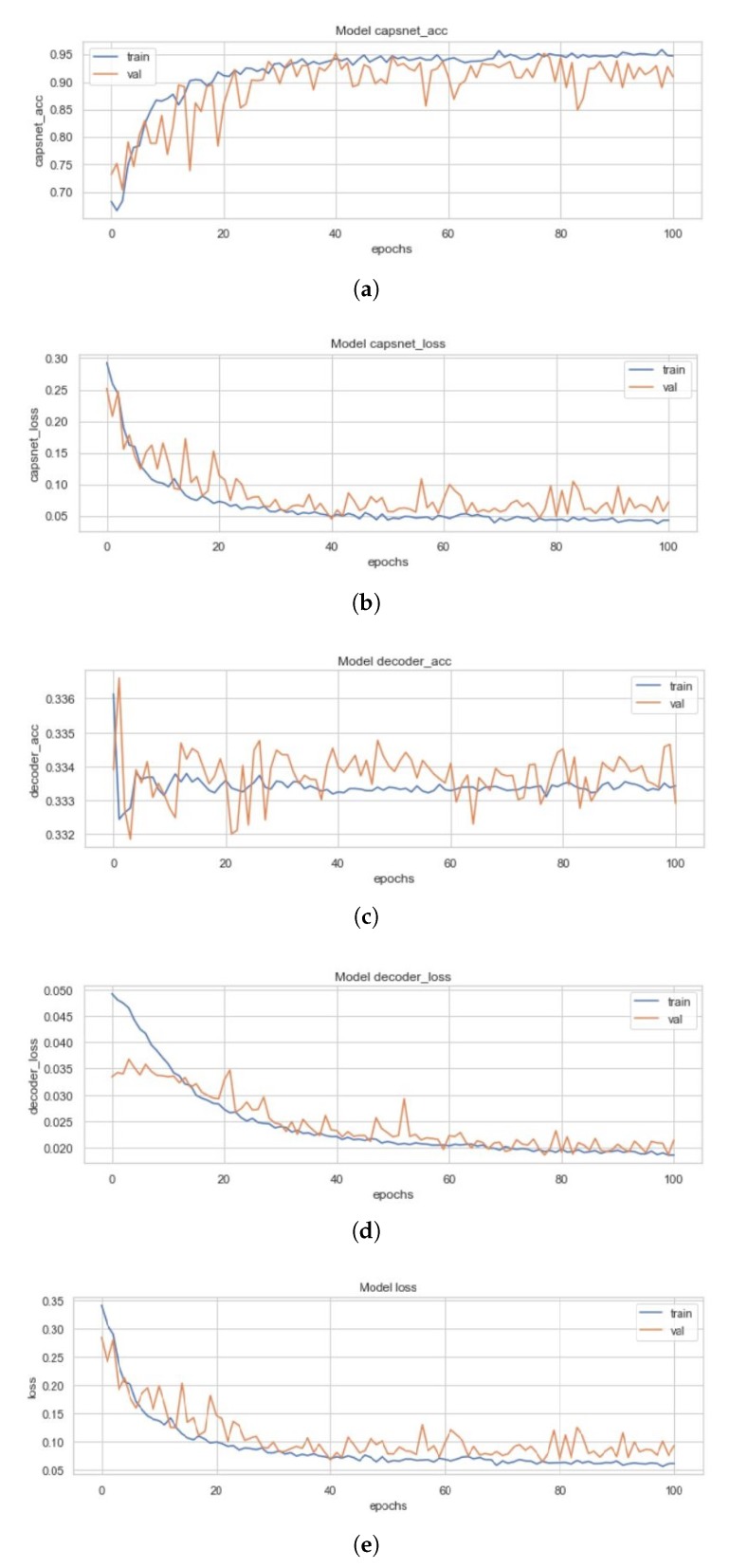
(**a**) Validation (in orange) and Training Accuracy (in blue) during the training of ICC model’s CapsNet. (**b**) Validation (in orange) and Training Loss (in blue) during the training of ICC model’s CapsNet. (**c**) Validation (in orange) and Training Accuracy (in blue) during the training of ICC model’s Decoder. (**d**) Validation (in orange) and Training Loss (in blue) during the training of ICC model’s Decoder. (**e**) Validation (in orange) and Training Loss (in blue) during the training of ICC model’s CapsNet + Decoder.

The DRS—the next part of E3CC—as earlier stated, has the Primary Caps layer, which has 32 channels of 8-D capsules. Each of these primary capsules has been provided with 192 × 9 units through ReLU Conv4. The Primary Caps have 32 × 17 × 17 outputs from the capsules where each output is 32-D (which is one of the differences between ECC and E3CC). Here, each capsule in 17 × 17 feature map behaves as explained earlier and the configuration for the capsules (i.e., their routing, their margin loss, etc.) remains as had been used in the ECC model. Conclusively, $Y_{out}$ has been used for detecting “Normal” or “Pneumonia” from CXR images. Similar to ECC, E3CC uses the decoder network (Figure 11b) for the same reason for encoding and regularizing activity instantiation vectors so they can be resistant and robust to pixel attacks. However, apart from their purpose to resist such attacks, they have not been optimized here to generate “Pneumonia” or “Normal” CXR images. Instead, they generate the image that has gone through the 3-convolution subnetworks in earlier E3-dCNS.

The EnCC model explained here, explains variants apart from the E3CC. These variants are named so, from the number of their convolutional subnetworks (En-dCNS). The normal ECC model (or E2CC) model can be generalized as the model that utilizes the least number of such subnetworks; therefore, it remains the most basic type of models among these defined variants. Here, n ∈ (4,8,16) as E3CC has been explained earlier. The EnCC model consists of 2 × n maxpool layers corresponding to 3 × n convolutions. These are divided among n ensembles of 3 convolutions and 2 maxpooling. The whole architecture of the model has been elucidated in Figure 9. The equations for nonlinearity functions and their derivatives have been defined in Figure A3 and Figure A4. The output from the e_1_ReLU Conv3, e_2_ReLU Conv3⋯, e_n_ReLU Conv3 is merged using the “concatenate” function.

The DRS—the next part of EnCC—as earlier stated, has the Primary Caps layer, which has 32 channels of 8-D capsules. Each of these primary capsules has been provided with 256 × 9 units through ReLU Conv4. The Primary Caps have 32 × 17 × 17 outputs from the capsules where each output is 32-D (which is one of the differences between ECC and EnCC). Here, each capsule in 17 × 17 feature map behaves as explained earlier and the configuration for the capsules (i.e., their routing, their margin loss, etc.) remains as had been used in the ECC model. Conclusively, $Y_{out}$ has been used for detecting “Normal” or “Pneumonia” from CXR images. Similar to ECC, EnCC uses a decoder network (Figure 9b) for the same reason of encoding and regularizing activity instantiation vectors. But apart from their purpose to regularize, they haven’t been optimized here to generate “Pneumonia” or “Normal” CXR images as they instead generate the image that has gone through the n-convolution subnetworks in earlier En-dCNS. The eLU and hyperbolic tangent (tanh) nonlinearity have been used in EnCC models (Figure 8 and Figure 9), and have been explained in Appendix A with Figure A3 and Figure A4.

## 4. Results

### 4.1. Simple CapsNet Model

All three models and the four variants of the ECC (i.e., the EnCC models) discussed above had been trained on a set of 4100 images, validated on 879 images and had been tested with 878 images. Figure 10 represents the training curves for the simple CapsNet. These figures are representative of the CapsNet accuracy, CapsNet loss, Decoder accuracy (As discussed earlier, the decoder is not able to generate back the images (signified by the low decoder accuracy and loss in Figure 10c,d that it got due to vector mapping limitations, there are only three plots (one for each Simple CapsNet, ICC and ECC) representing the training of decoder.), Decoder loss, and the total loss (sum of all losses) from Figure 10a–e) for simple CapsNet, in order. All these figures had been created using the history of training from simple CapsNet. The packages used were Matplotlib [38], Keras [39], and Seaborn. This model converged after 161 epochs and had CapsNet validation accuracy of 93.75% (therefore being comparable to model in [30]). The other values have also been listed in Table 3 below.

### 4.2. ICC Model

Figure 11 represents the training curves for the ICC model described earlier. These figures are representative of the CapsNet accuracy, CapsNet (Note that the CapsNet accuracy and all other terms mentioned here have CapsNet, because the whole architecture having capsule layers is usually divided into 2 different networks which can help encoding of data through activity instantiation vector. Therefore, “simple CapsNet” discussed above and the “CapsNet accuracy” discussed here and later onwards does not refer to the first model’s accuracy. Rather it is the accuracy of the discriminative model, and term “Decoder accuracy” is generative model’s accuracy.) loss, Decoder accuracy, Decoder loss, and the total loss (sum of all losses) from Figure 11a–e for the ICC model, in order. All these figures had been created using the history of training from ICC. This model converged after 100 epochs and had CapsNet validation accuracy of 95.22%. The other values have also been listed in Table 3. These 128 feature maps of convoluted images are then reconstructed through the capsule layers, which when compared to the input image (pixel-wise) has 33.34% (decoder accuracy) similarity as the feature maps of convoluted images are of dimensions 8 × 8.

### 4.3. ECC Model

**Figure 12:**
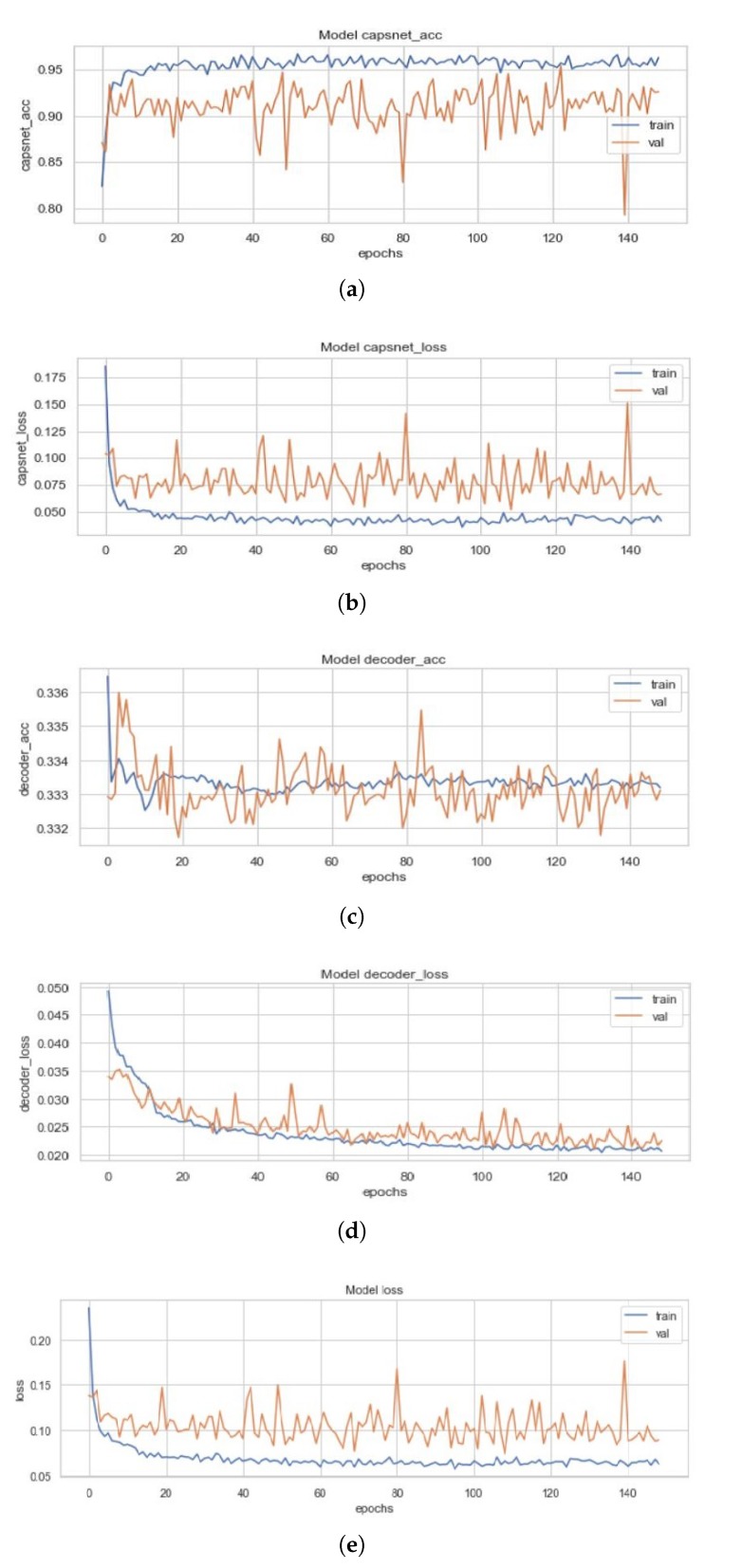
(**a**) Validation (in orange) and Training Accuracy (in blue) during the training of ECC model’s CapsNet. (**b**) Validation (in orange) and Training Loss (in blue) during the training of ECC model’s CapsNet. (**c**) Validation (in orange) and Training Accuracy (in blue) during the training of ECC model’s Decoder. (**d**) Validation (in orange) and Training Loss (in blue) during the training of ECC model’s Decoder. (**e**) Validation (in orange) and Training Loss (in blue) during the training of ECC model’s CapsNet + Decoder.

Figure 12 represents the training curves for the ECC model described earlier. These figures are representative of the CapsNet accuracy, CapsNet loss, Decoder accuracy (Training curves for EnCC (where n = 3, 4, 8, 16) will not be depicted as they reconstruct the convoluted images rather than the actual images but the intensities are compared with the actual image.), Decoder loss, and the total loss (sum of all losses) from Figure 12a–e for ECC, in order. All these figures had been created using the history of training from ECC. This model converged after 149 epochs and had CapsNet validation accuracy of 95.41% (just slightly higher than that of the ICC model). The other values have also been listed in Table 3. This model used two subnetworks of convolutions with the Dynamic Routing Subnetwork. These two subnetworks each contribute 128 feature maps of convoluted images, bringing the total feature maps to 256. These concatenated 256 feature maps of convoluted images are then reconstructed through the capsule layers, which when compared to the input image (pixel-wise) has 33.32% (decoder accuracy) similarity as the feature maps of convoluted images are of dimensions 19 × 19.

### 4.4. EnCC Model

**Figure 13:**
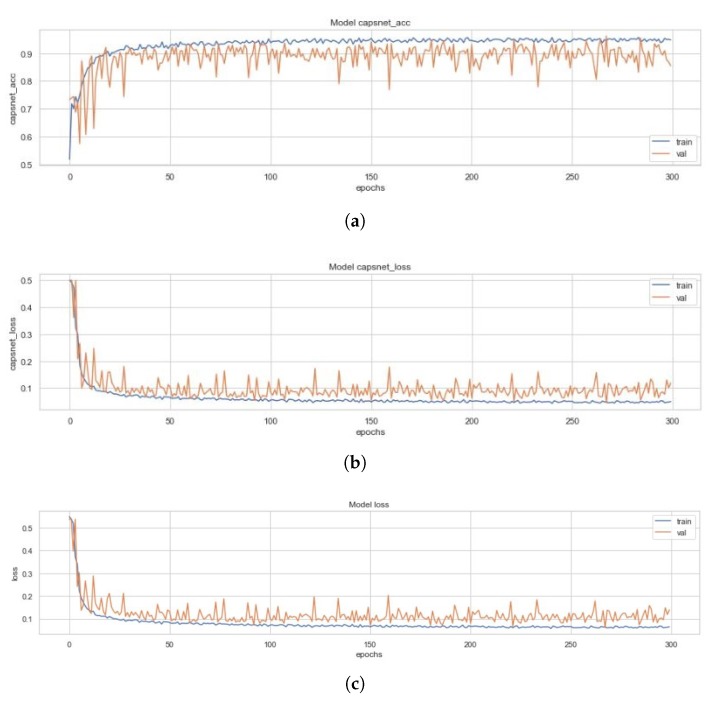
(**a**) Validation (in orange) and Training Accuracy (in blue) during the training of E4CC model’s CapsNet. (**b**) Validation (in orange) and Training Loss (in blue) during the training of E4CC model’s CapsNet. (**c**) Validation (in orange) and Training Loss (in blue) during the training of E4CC model’s CapsNet + Decoder.

Figure 13 represents the training curve for the E4CC model described earlier. These figures are representative of the CapsNet accuracy, CapsNet loss, and the total loss (sum of all losses) from Figure 13 for E4CC. All these figures have been created using the history of training from EnCC. The training curves for E3CC, E8CC, and E16CC have been depicted in the Appendix B as they do not give accuracy as high as E4CC variant. The model E4CC converged after 300 epochs and had CapsNet validation accuracy of 96.29% (which is the best amongst ECC and its variants). The other values have also been listed in Table 3. This model used four subnetworks of convolutions with the Dynamic Routing Subnetwork. These four subnetworks each contribute 64 feature maps of convoluted images, bringing the total feature maps to 256. And these concatenated 256 feature maps of convoluted images are then reconstructed through the capsule layers, which when compared to the input image (pixel-wise) has 33.38% (decoder accuracy) similarity as the feature maps of convoluted images are of dimensions 19 × 19. Therefore, according to the validation accuracy and decoder accuracy, the variant of the ECC model (named E4CC) detects Pneumonia most accurately, while encoding and regularizing the convolutional and capsule layers the best using a decoder network that had been defined in Figure 9b. The following section depicts the testing results of all the models along with their confusion matrices. The test set, as mentioned in Section 3.1, consists of 878 images, of which 641 are “Pneumonia” and 237 are “Normal” images.

### 4.5. Test Results

**Figure 14:**
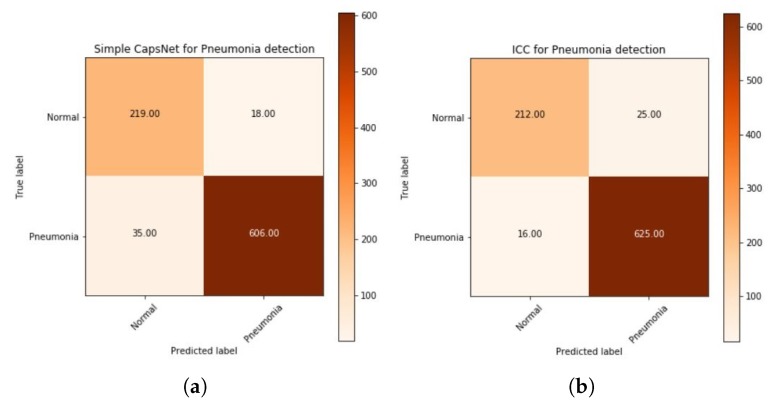

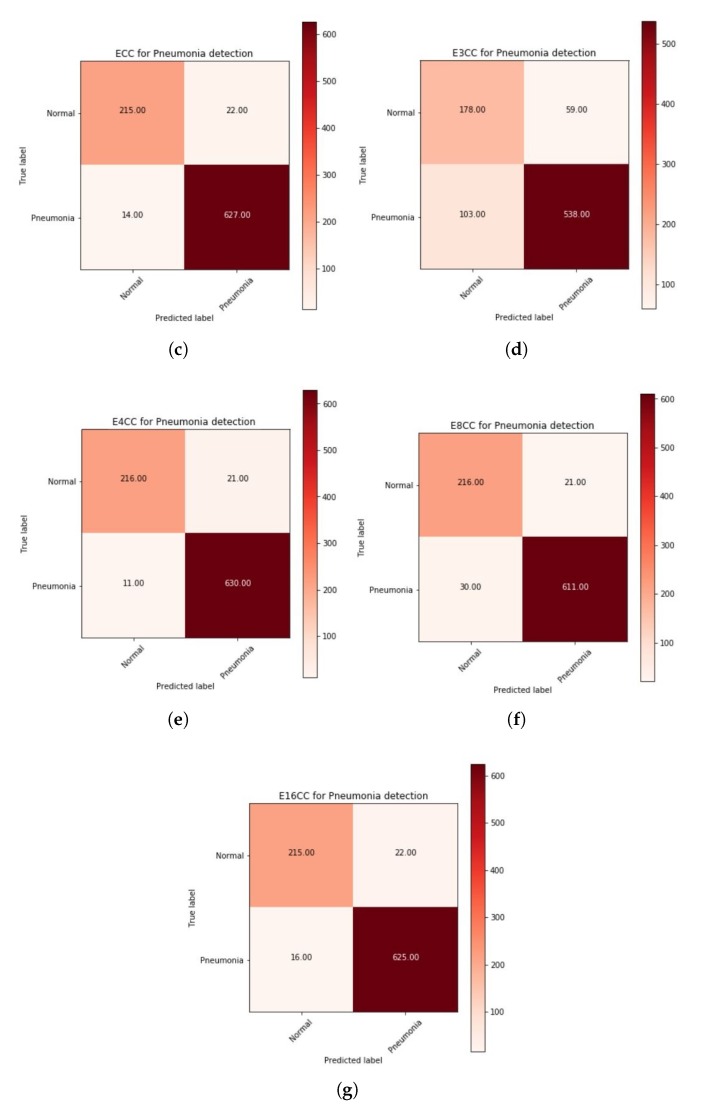
Plot of confusion matrix for detection of Pneumonia using (**a**) Simple CapsNet, (**b**) Integration of convolutions with capsules (ICC), (**c**) Ensemble of convolutions with capsules (ECC), (**d**) Ensemble of 3-convolutions with capsules (E3CC), (**e**) Ensemble of 4-convolutions with capsules (E4CC), (**f**) Ensemble of 8-convolutions with capsules (E8CC), and (**g**) Ensemble of 16-convolutions with capsules (E16CC).

The plot of a confusion matrix for all three of the models whose training history has been mentioned earlier in Figure 10, Figure 11 and Figure 12 for simple CapsNet, ICC, and ECC, respectively, have been given in Figure 14. It can be observed that the simple CapsNet model (Figure 14a) has 35 false negatives (FNs) (5.46% of “Pneumonia” CXR images) from the CXR test set of 878 images. In pneumonia detection, FNs can prove to be fatal as someone who has pneumonia may be denied of its possibility and therefore, may get the wrong treatment for it. On the other hand, this model has 18 false positives (FPs) (7.59% of “Normal” CXR images), which have otherwise been normal CXR images predicted to have pneumonia.

The ICC model, discussed earlier has its confusion matrix in Figure 14b. It classifies 625 instances of pneumonia positively (97.5%) and has 16 false negatives (FNs) (2.5% of “Pneumonia” CXR images) from the CXR test set of 878 images. On the other hand, this model has 25 false positives (FPs) (10.55% of “Normal” CXR images), which have otherwise been normal CXR images predicted to have pneumonia. It may be observed that although the amount of FNs decrease, the number of FPs have increased but FPs may not be as harmful as FNs. This is because someone who does not have pneumonia and has got a positive test would be subjected to medications that would otherwise only get rid of pneumonia. Therefore, the amount of FPs is acceptable within a certain scope until and unless patients are not suffering from different diseases. A more detailed study should be conducted if the aforementioned case is true.

The plot of the confusion matrix for ECC has been given in Figure 14c. This model has further improved the test accuracy of the Pneumonia dataset and has also led to reducing the number of FNs to 14 (i.e., 2.18% of “Pneumonia” CXR images) and 22 false positives (FPs) (10.55% of “Normal” CXR images) It also has less misclassified points, better CapsNet validation accuracy compared to simple CapsNet model and ICC model as has been discussed in regards to Table 3.

Finally, the plots of the confusion matrix for variants of EnCC models have been given in Figure 14d–g. Among these models, E4CC (Figure 14e) has further improved (from ECC model) the test accuracy of the Pneumonia dataset and has also led to reducing the number of FNs to 11 (i.e., 1.71% of “Pneumonia” CXR images) and 21 false positives (FPs) (8.86% of “Normal” CXR images) It also has less misclassified points, better CapsNet validation accuracy compared to simple CapsNet model, ICC model, ECC and E16CC (which has 16 FNs and 22 FPs) as has been discussed in regards to Table 3. A detailed comparison of all three models and all variants of the ECC model that have been implemented has been done in Table 3, which has been given below. Note that E4CC has been the best model among the models mentioned here. This has been because four convolution subnetworks compute 64 feature maps to be given to the concatenation layer, which is then routed through the capsules, as discussed earlier in Section 3. The calculation of features from both the convolutions make E4CC more robust and increases its equivariance to spatial correlation.

An exegesis of the results suggests that the ICC model converges the fastest for the dataset described in Section 3.1. Despite this, and its low value of loss (CN Loss$t_{r}$), it has a comparatively lower accuracy (CN Acc$t_{r}$, CN Acc$v_{a}$, and CN Acc$t_{e}$). Here, we can see that the best models in terms of accuracy (CN Acc$t_{r}$, CN Acc$v_{a}$, and CN Acc$t_{e}$) is the E4CC model. Apart from this, a mention of Decoder Accuracy (D Acc$t_{r}$ and D Acc$v_{a}$) and Loss (D Loss$t_{r}$ and D Loss$v_{a}$) is necessary over here. Note that this work has been a classification task and therefore primarily used the decoder for encoding and regularizing the activity instantiation vector, which is then sent back through receptive fields of the capsule layer above the connected capsule layer. However, in most of these models, except Simple CapsNet, the decoder has been used to reconstruct the image and regularize and encode the convoluted feature maps obtained by the primary capsule layer in all these models. The results have further been discussed and conclusions have been drawn from Table 3.

## 5. Discussions and Conclusions

The models introduced in this research, ICC, ECC, and its two best variants (E4CC and E16CC), achieved a training accuracy (CN Acc$t_{r}$) of 94.29%, 95.70%, 96.36%, and 95.67% and training loss (CN Loss$t_{r}$) of 0.048, 0.043,0.049, and 0.057, respectively, on the set of 4100 images. These models had been robust to overfitting and pixel attacks, with the help of decoder and achieved a validation accuracy (CN Acc$v_{a}$) of 95.22%, 95.41%, 96.29%, 95.30%, and validation loss (CN Loss$v_{a}$) of 0.045, 0.062, 0.045, and 0.054 on a set of 879 images for ICC and ECC, respectively. These models have been tested on 878 images and achieved an accuracy (Acc$t_{e}$) of 95.33%, 95.90%, 96.36%, and 95.67% as has been observed from confusion matrices in Figure 14b,c,e,g, respectively. All these models combined convolution operations with dynamic capsule routing to achieve the aforementioned results, the only difference being that one model combined one set of convolutions, while the others combined two or more sets of convolutions. All of these models (simple CapsNet, ICC, ECC, and ECC variants) had been trained on the ratio of 14:3:3 for train, validation, and a test sets of images (with 5857 being the total number of images).

Although all these models achieved higher testing and validation accuracy and lower validation loss compared to the models mentioned earlier in Table 1, they still suffer from some drawbacks. This has been ascribed to the fact that all these models are not able to efficiently utilize their generative capabilities to produce accurate computer-generated images (CGIs) for CXR from the given set of images, even though the generative capabilities of the models lie outside the scope of this research. This work can further be extended through the utilization of a more robust generative model (or decoder) to generate high-quality images for CXR to get better generative capabilities. Some more areas where this work can be extended have been discussed below. ICCs and ECCs used convolutions and capsules that had been generated through the trial-and-error method, which is not an efficient means for defining a good classification or generative model. Note that genetic algorithms can be employed to design an optimal blend of convolutions for classification purposes [47,48,49]. Along with this, the generation of optimal capsule layers can also be secured through a genetic algorithm [50,51] (just as in the case of DNNs) or other evolutionary strategies [52,53]. This work can also utilize Edge detection techniques [54] for additional parameters which can be fed to the ECC model proposed.

## Figures and Tables

**Table 1:** Comparative analysis of various deep learning models in accordance to the literature.

Models	Accuracy Achieved
CheXNet [23]	76.80%
Can J Saul et al. [24] CNN	78.83%
Xception pretrained model	82%
VGG-16 pretrained model [25]	87%
VGG-19 pretrained model CNN	92%
R H Abiyev et al. [29] CNN	92.40%
Okeke Stephan et al. [30] CNN	93.73%
ResNet-152	88%
VGG-16 pretrained model	88.89%
A Sagar CNN	91.02%
J Erthal CNN	80%
ResNet-34	91%
VGG-16 + Capsnet	88.30% (after 10 epochs)

**Table 2:** A complete distribution of pneumonia CXR images into train, validation, and test set.

Classes	Train Set	Validation Set	Test Set	Total
Normal	1108	238	237	1583
Pneumonia	2992	641	641	4274

**Table 3:** A comprehensive comparison of different models used for the detection of Pneumonia from CXR images. These algorithms have been compared on basis of no. of epochs at which model converged, CapsNet accuracy (CN Acc$t_{r}$, CN A${\mathrm{cc}}_{valid}$, CN Acc$T_{e}$), CapsNet loss (CN Loss$t_{r}$, CN Loss$v_{a}$), Decoder accuracy (D Acc$t_{r}$, D Acc$v_{a}$), Decoder loss (D Loss$t_{r}$, D Loss$v_{a}$), and total loss (sum of all losses) (Loss$t_{r}$, Loss$t_{r}$). Here, CapsNet in the first row is the Simple CapsNet model discussed in this work.

Metrics	CapsNet	ICC	ECC	E3CC	E4CC	E8CC	E16CC
epochs	162	100	148	213	300	182	190
CN Acc$t_{r}$	90.25%	94.29%	95.70%	71.78%	95.53%	95.36%	94.19%
CN Acc$v_{a}$	93.75%	95.22%	95.41%	77.69%	96.29%	94.53%	95.30%
Acc$t_{e}$	93.96%	95.33%	95.90%	81.54%	96.36%	94.19%	95.67%
CN Loss$t_{r}$	0.089	0.048	0.043	0.186	0.049	0.050	0.057
CN Loss$v_{a}$	0.086	0.045	0.062	0.1979	0.045	0.063	0.054
D Acc$t_{r}$	6.63%	33.32%	33.33%	33.39%	33.33%	33.35%	33.32%
D Acc$v_{a}$	4.91%	33.34%	33.32%	33.25%	33.38%	33.36%	33.29%
D Loss$t_{r}$	0.017	0.022	0.021	0.023	0.015	0.015	0.017
D Loss$v_{a}$	0.019	0.023	0.022	0.028	0.016	0.019	0.021
Loss$t_{r}$	0.106	0.070	0.065	0.2094	0.064	0.065	0.074
Loss$v_{a}$	0.105	0.068	0.087	0.226	0.061	0.082	0.075

**Figure A5:**
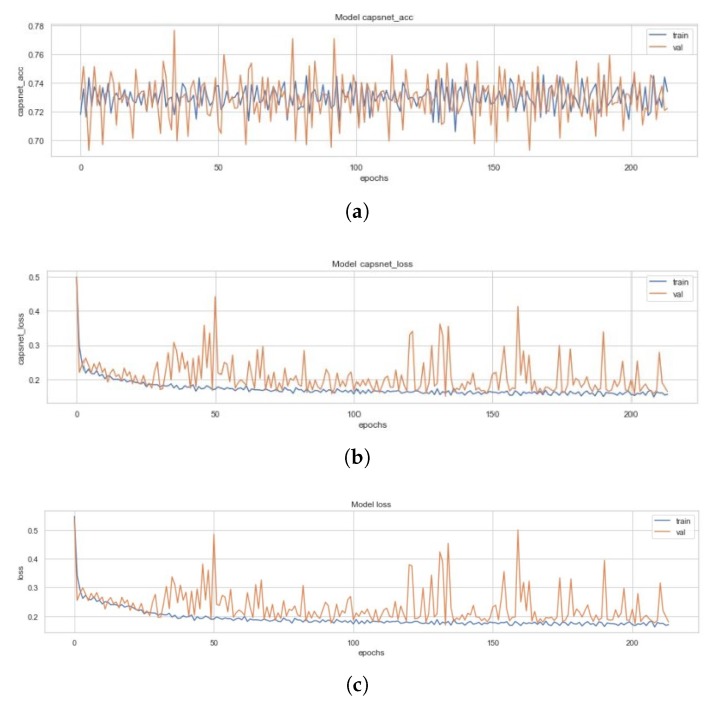
(**a**) Validation (in orange) and Training Accuracy (in blue) during the training of E3CC model’s CapsNet. (**b**) Validation (in orange) and Training Loss (in blue) during the training of E3CC model’s CapsNet. (**c**) Validation (in orange) and Training Loss (in blue) during the training of E3CC model’s CapsNet + Decoder.

**Figure A6:**
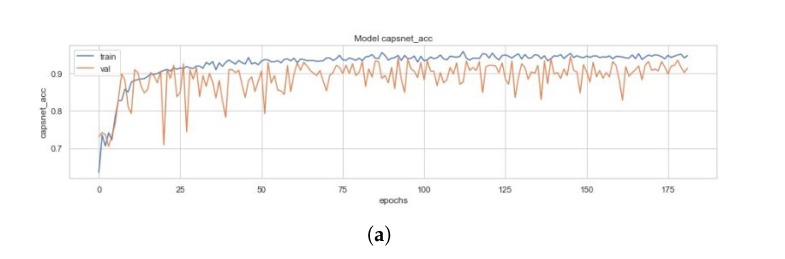

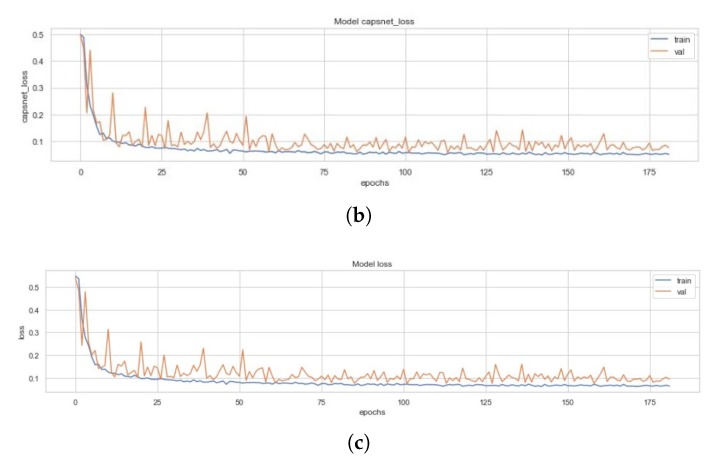
(**a**) Validation (in orange) and Training Accuracy (in blue) during the training of E8CC model’s CapsNet. (**b**) Validation (in orange) and Training Loss (in blue) during the training of E8CC model’s CapsNet. (**c**) Validation (in orange) and Training Loss (in blue) during the training of E8CC model’s CapsNet + Decoder.

**Figure A7:**
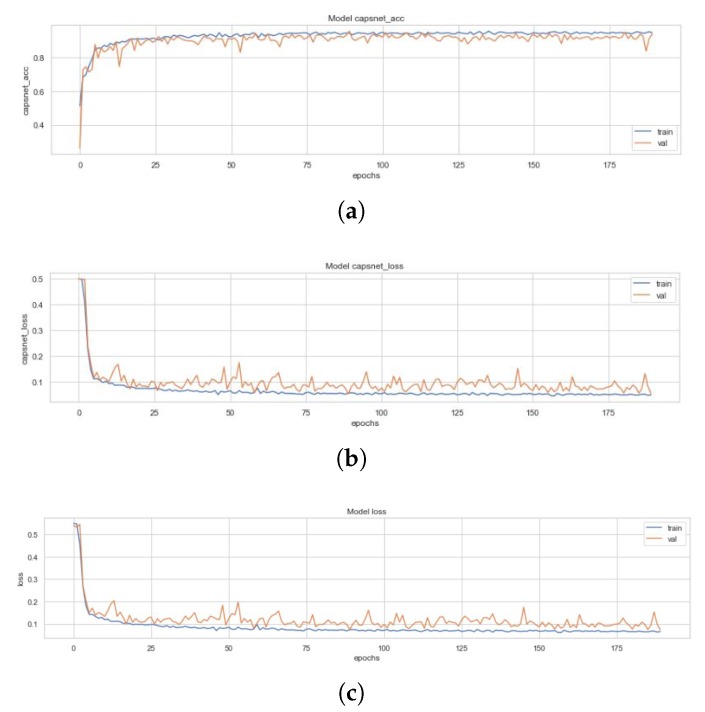
(**a**) Validation (in orange) and Training Accuracy (in blue) during the training of E16CC model’s CapsNet. (**b**) Validation (in orange) and Training Loss (in blue) during the training of E16CC model’s CapsNet. (**c**) Validation (in orange) and Training Loss (in blue) during the training of E16CC model’s CapsNet + Decoder.

## Appendix A. Activation Functions Used

This section deals with all the activation functions that had been used in the models that have been discussed in the Section 3.3. These functions have been described along with their derivatives which will help the readers to better understand the nuances of applications of these functions. The first function we discuss is the Rectified Linear Unit function, which is also commonly known as ReLU function.

(A1) $$ReLU\left(z\right)=\left\{\begin{array}{c} z,\;\mathrm{if}\;z\geq 0\\ 0,\;\mathrm{if}\;z<0 \end{array}\right.$$

As has been explicable from the equation, the ReLU function uses the half rectified output to introduce nonlinearity to local features. From Equation (A1), it can be seen that feature vectors with negative values are nullified while the positive local features are retained. It and its derivatives are monotonic in nature and it has therefore, been used while routing the feature vectors from convolutions to the initial capsule layer. The equation for its derivative has been given in Equation (A2).

(A2) $$ReLU^{\prime }\left(z\right)=\left\{\begin{array}{c} 1,\;\mathrm{if}\;z\geq 0\\ 0,\;\mathrm{if}\;z<0 \end{array}\right.$$

In Equations (A1) and (A2), *z* demonstrates the local feature vector that has been extracted from the local pixel intensity values from CXR images. Figure A1a,b demonstrates the monotonicity of both the ReLU function and its derivative. This means that both the functions only increase or remain same when local feature vectors increase, and decrease or remain the same when the vectors’ values decrease.

The next function that had been used is given in Equation (A3) which has been explained briefly for the context of this application.

(A3) $$\sigma \left(z\right)=\frac{1}{1+e^{-z}}$$

This function takes all real values as inputs and outputs values between 0 and 1. It asymptotes to 0 and 1 for values of –∞ and ∞, respectively. At *z* = 0, this function gives a value of 0.5. The function has been depicted in Figure A2a. It is an S-shaped curve that introduces no binary activations (as those in step functions), and therefore remains one of the most prominent nonlinearity for binary classification (here, “Normal” or “Pneumonia” CXR images). Here, the sigmoid gives a probability pixel for each masked vector values (augmentation of activity instantiation vector and class of that vector). This function is monotonic, but, its derivative is not monotonic as has described in Equations (A3) and (A4) and Figure A2.

(A4) $$\sigma^{\prime }\left(z\right)=\sigma \left(z\right)\cdot \left(1-\sigma \left(z\right)\right),\;\mathrm{or}\;\frac{e^{-z}}{{\left(1+e^{-z}\right)}^{2}}$$

In Equations (A3) and (A4), *z* demonstrates the local feature vector that had been extracted from the local pixel intensity values from CXR images. Figure A2a,b demonstrates the monotonicity and non-monotonicity of the sigmoid and its derivative, respectively. The next nonlinearity that we deal with is given in Equation (A5) which has again been explained with reference to the context of the application of Pneumonia Detection.

(A5) $$eLU\left(z\right)=\left\{\begin{array}{c} z,\;\mathrm{if}\;z\geq 0\\ a\cdot (e^{z}-1),\;\mathrm{if}\;z<0 \end{array}\right.$$

The eLU function is the same as ReLU. It slowly smoothens until its output equals −1 (here, *a* = 1). The eLU function tends to account for negative pixel intensities, and therefore can be comparatively better than ReLU. Its derivative is given in Equation (A6). The eLU function and its derivatives are monotonic and differentiable. The eLU function has been depicted in Figure A3a, while its derivative has been given in Figure A3b.

(A6) $$eLU^{\prime }\left(z\right)=\left\{\begin{array}{c} 1,\;\mathrm{if}\;z\geq 0\\ a\cdot e^{z},\;\mathrm{if}\;z<0 \end{array}\right.$$

The tanh (hyperbolic tangent) nonlinearity has been used in EnCC and decoder modules of variants defined in Figure 8 and Figure 9). It has been explained using the Equation (A7) in the context of Pneumonia Detection.

(A7) $$tanh\left(z\right)=\frac{e^{z}-e^{-z}}{e^{z}+e^{-z}}$$

where *z* is considered as the output values of pixels from the previous layer of the Convolutional Neural Network for individual images. This function creates an S-shaped curve (as shown in Figure A4a) due to its exponential (i.e., ez or e-z) terms. This is because of the function defined above asymptotes at −1 and +1 as the value of *z* increases or decreases, respectively. Its derivative is given in Equation (A8). It is monotonic and differentiable, whereas its derivative is not. The tanh function has been depicted in Figure A4a, whereas its derivative has been given in Figure A4b.

(A8) $$tanh^{\prime }\left(z\right)=\frac{4}{{\left(e^{z}+e^{-z}\right)}^{2}}$$

## Appendix B. Training Curves for Model Variants

The code and outputs pertaining to this paper have been uploaded on the GitHub repository: AnshMittal1811/PneumoniaDetection. This section depicts the training of the E3CC, E8CC, and E16CC variants of the ECC model, corresponding to the CapsNet accuracy, CapsNet loss, and the total loss of the model variants, respectively. These have been depicted in the following. Figure A5 represents the training curve for the E3CC model described earlier. These figures are representative of the CapsNet accuracy, CapsNet loss, and the total loss (sum of all losses) from Figure A5a–c for E3CC, in order. All these figures had been created using the history of training from E3CC. This model converged after 213 epochs and had CapsNet validation accuracy of 77.69% (which is the worst among ECC and its variants). The other values have also been listed in Table 3. This model used three subnetworks of convolutions with the Dynamic Routing Subnetwork. These three subnetworks each contribute 64 feature maps of convoluted images, bringing the total feature maps to 192, and these concatenated 192 feature maps of convoluted images are then reconstructed through the capsule layers, which when compared to the input image (pixel-wise) has 33.25% (decoder accuracy) similarity as the feature maps of convoluted images are of dimensions 19 × 19.

The variant E8CC converged after 182 epochs and had CapsNet validation accuracy of 94.53%. The other values have also been listed in Table 3. This model used eight subnetworks of convolutions with the Dynamic Routing Subnetwork. This model used eight subnetworks of convolutions with the Dynamic Routing Subnetwork. These eight subnetworks each contribute 32 feature maps of convoluted images, bringing the total feature maps to 256, and these concatenated 256 feature maps of convoluted images are then reconstructed through the capsule layers, which when compared to the input image (pixel-wise) has 33.36% (decoder accuracy) similarity as the feature maps of convoluted images are of dimensions 19 × 19.

The variant E16CC converged after 190 epochs and had CapsNet validation accuracy of 95.303% (which is just below the ECC model). The other values have also been listed in Table 3. This model used 16 subnetworks of convolutions with the Dynamic Routing Subnetwork. These 16 subnetworks each contribute 16 feature maps of convoluted images, bringing the total feature maps to 256, and these concatenated 256 feature maps of convoluted images are then reconstructed through the capsule layers, which when compared to the input image (pixel-wise) has 33.29% (decoder accuracy) similarity as the feature maps of convoluted images are of dimensions 19 × 19.

## Abbreviations

The following abbreviations are used in this manuscript:

CNN	Convolutional Neural Networks
CXR	Chest X-Ray
ROI	Region Of Interest
ICC	Integration of Convolutions with Capsules
ECC	Ensemble of Convolutions with Capsules
EnCC	Ensemble of n-Convolutions with Capsules
